# Association of *TLR4* and *TLR9* gene polymorphisms with cervical HR-HPV infection status in Chinese Han population

**DOI:** 10.1186/s12879-023-08116-z

**Published:** 2023-03-13

**Authors:** Chunlin Zhang, Zhiping Yang, Ping Luo, Ting Li, Sutong Wang, Fenglan Sun, Ping Gong, Bing Mei

**Affiliations:** 1 Department of Laboratory Medicine, Jingzhou Hospital Affiliated to Yangtze University, Jingzhou, 434020 Hubei China; 2 Department of Pathology, Jingzhou Hospital Affiliated to Yangtze University, Jingzhou, 434020 Hubei China

**Keywords:** Persistent HR-HPV infection, Multiple HR-HPV infections, Toll-like receptors, Single nucleotide polymorphisms

## Abstract

**Background:**

Toll-like receptors (TLRs) may be involved in the natural history of human papillomavirus (HPV) infection. In our study, we aimed to investigate the association of *TLR4* (rs10116253, rs1927911, rs10759931) and *TLR9* (rs187084, rs352140) gene polymorphisms with cervical persistent high-risk HPV (HR-HPV) infection, as well as multiple HR-HPV infections.

**Methods:**

A total of 269 study subjects were enrolled and grouped by retrospectively analyzing the HR-HPV testing results and other clinical data of 2647 gynecological outpatients from Jingzhou Hospital Affiliated to Yangtze University. We conducted a case–control study to compare the role of *TLR4/TLR9* gene polymorphisms between HR-HPV transient and persistent infections, as well as between HR-HPV single and multiple infections. HR-HPV genotypes were detected using Real-time polymerase chain reaction (RT-PCR). PCR-restriction fragment length polymorphism (PCR–RFLP) was used to determine *TLR4* and *TLR9* gene polymorphisms. Analyses of the different outcome variables (HR-HPV infection status and time for HR-HPV clearance) with respect to *TLR4/TLR9* polymorphisms were carried out. Logistic regression analysis was used to determine the association of *TLR4/TLR9* genotypes and alleles with HR-HPV infection status. The Kaplan–Meier method with the log-rank test was used to analyze the relationship between *TLR4/TLR9* genotypes and the time for HR-HPV clearance.

**Results:**

The mutant genotypes of *TLR9* rs187084 and rs352140 were associated with persistent (rs187084: CT and CT+CC; rs352140: CT and CT+TT) and multiple (rs187084: CT and CT+CC; rs352140: CT+TT) (all *P* < 0.05) HR-HPV infection. However, no association was found between *TLR4* polymorphisms and HR-HPV infection status. Kaplan–Meier time to HR-HPV clearance analysis demonstrated that women carrying rs187084 and rs352140 mutant genotypes take longer duration to clear HR-HPV infection compared with wild-type genotype carriers (*P*1 = 0.012; *P*2 = 0.031).

**Conclusion:**

Our results suggested that *TLR9* polymorphisms, but not *TLR4*, were associated with cervical persistent and multiple HR-HPV infections, which could be useful as a potential predictor of HR-HPV infection status.

**Supplementary Information:**

The online version contains supplementary material available at 10.1186/s12879-023-08116-z.

## Background

Human papillomaviruses (HPV) are the most common sexually transmitted pathogens worldwide. More than 200 HPV genotypes have been identified so far [1, 2]. High-risk human papillomavirus (HR-HPV), mainly including HPV types 16, 18, 31, 33, 35, 39, 45, 51, 52, 56, 58, 59, 66, and 68, are the definite risk factor for cervical intraepithelial neoplasia (CIN) and cervical cancer (CC) [1]. Nearly 99.7% of CC was caused by persistent HR-HPV infection [3]. Although the high prevalence of HR-HPV infections is shown among women all over the world, most infections are transient. A meta-analysis in 2013 combined data on over 100,000 women from 86 studies examining HPV persistence patterns worldwide. It reported that the weighted median duration of HR-HPV persisted was 9.3 months, estimates of the proportion persistent ranged from 18 to 90% in the 6th month and 24–63% in the 12th month for HR-HPV [4]. In several studies conducted in China, the average duration of persistent HR-HPV infection was 12.8 months, and the average proportion of persistent HR-HPV infection was 36.2% in the 24th month [5–7].

Toll-like receptors (TLRs) are belong to the family of pattern recognition receptors (PRRs), of which TLR9 is located intracellularly and recognizes unmethylated cytosine-phosphate-guanine (CPG) DNA, TLR4 is located at the cell membrane and mainly recognizes lipopolysaccharide (LPS) from bacteria and certain viral proteins [8–10]. TLRs play an important role in HPV clearance by stimulating innate and adaptive immune responses [11–14]. The interplay between host immune system and HPV are considered instrumental in shaping the natural history of HPV infection [15]. It was reported that high expression levels of TLR9 in the cervical mucosal were critical for HPV16 clearance [16]. Meanwhile, other study reported that HPV16 suppressed immunity by promoting the formation of an inhibitory transcriptional complex containing NF-κBp50–p65 and ERα, which resulted in decreased TLR9 expression [17]. In addition, as the coat proteins encoded by different HPV subtypes often harbor large genetic variations and cross-protective antibodies are absent between them, the host is susceptible to multiple HPV infections [18]. Study reported that multiple HPV infections were associated with persistent HPV infection [19].

Functional analyses have shown that *TLR4* rs10759931 and rs10116253 could be a binding site for GATA2 and NFATC2, respectively [20, 21]. In addition, Kikuchi et al. [22] observed that genetic polymorphisms of *TLR9* influenced the immune response to CpG, their further research found *TLR9* rs352140 TT genotype enhanced gene expression and was associated with higher frequency of intracellular IgM + B cells. Therefore, TLRs gene mutations may alter the expression or function of the corresponding encoded proteins, and affect the individual immune responses. The majority of the previous studies have focused on the association between *TLR4/TLR9* single nucleotide polymorphisms (SNPs) and HPV susceptibility [23–26]. The rs187084 TT genotype, rs352140 TT/CT genotype and rs1927911 CT genotype were reported to increase the risk of HPV infection [23, 24]. However, the role of the *TLRs* SNPs in the history of HPV infection were poor reported. In the Ludwig–McGill Cohort Study, Oliveira et al. [27] investigated the role of *TLR9* rs5743836 in clearance/persistence of type-specific HPV infection and no association was found. There only one study conducted in India reported *TLR4* 10759931 AG/GG genotype showed protective effect against the risk of acquiring multiple HR-HPV infections [28].

Based on the important role of TLRs in antiviral immune response, the aim of this study was to investigate the association of *TLR4* (rs10116253, rs1927911, rs10759931) and *TLR9* (rs187084, rs352140) gene polymorphisms with cervical HR-HPV infection status.

## Materials and methods

### Study subjects

**Fig. 1:**
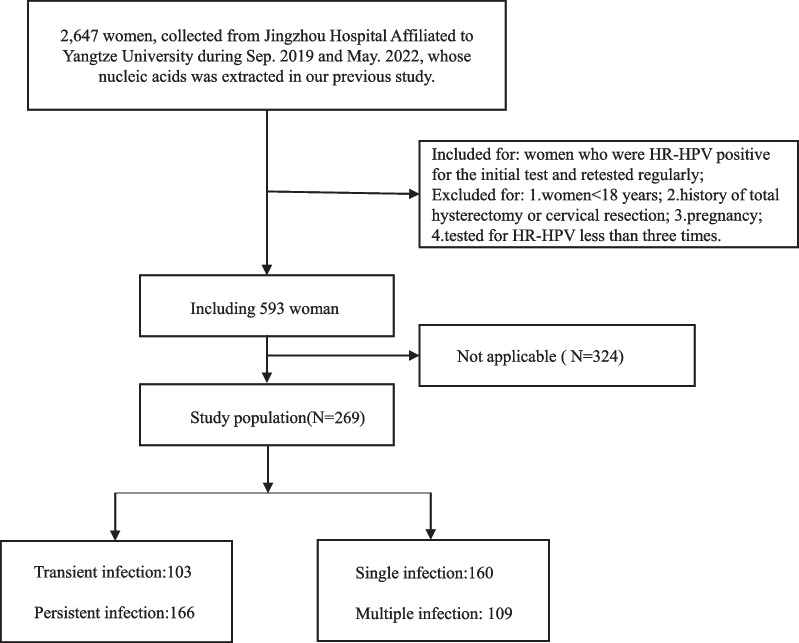
Flow chart of participants’ inclusion and exclusion

In this case–control study, subjects were collected from 2647 women who tested for HR-HPV at the gynecological outpatient clinic of Jingzhou Hospital Affiliated to Yangtze university from Sep. 2019 to May. 2022, whose genomic DNA was extracted in our previous study. The HR-HPV testing results and clinical data for these 2647 patients were obtained from laboratory information system (LIS) and outpatient electronic medical records (EMRs), respectively. According to the inclusion and exclusion criteria, there were 593 patients were enrolled in the retrospective cohort to estimate individual HR-HPV infection status. Inclusion criteria: women who were HR-HPV positive for the initial testing and retested regularly within the following 36 months. Exclusion criteria: age < 18; pregnancy; history of total hysterectomy or cervical resection; tested for HR-HPV less than 3 times. Ultimately, 269 patients were enrolled in the study and grouped according to the definition of HR-HPV infection status, as transient HR-HPV infection, persistent HR-HPV infection, single HR-HPV infection, and multiple HR-HPV infection. The flow chart for study subject collection was shown in Fig. 1.

DNA was extracted from exfoliated cervical cells according to the instruction of the nucleic acid extracting kit (Shanghai ZJ Bio‐Tech Co., Ltd. and Guangzhou Magen Biotechnology Co., Ltd.) and stored at − 80 °C for use, as our previous studies described in detail [29–31]. This study was approved by the Ethics Committee of the Jingzhou Hospital Affiliated to Yangtze University (Code: 2022-048-01). All research was performed in accordance with relevant guidelines. Informed consent of patients had been obtained, and the study subjects’ privacy was protected before sample collection.

### Definition of HR-HPV infection status

There were wide variations in the definition of HPV persistence used in literatures. In most studies, HPV persistence was defined as the detection of the same HPV genotype or the same type-group in two consecutive visits [19, 27, 32]. A meta-analysis conducted by Rositch et al. [4] showed that the minimum duration of HPV persistence, defined as the shortest time period of HPV positivity for a woman to be considered persistent, was 6–12 months for approximately half of studies. Throughout all studies, the median duration of high-risk HPV infection was 9.3 months. Therefore, we clarify HR-HPV infection status with the results of HR-HPV testing at certain time intervals. We divided 36 months into 3 calendar periods: within 12 months, 12–24 months, 24–36 months, after the HR-HPV positive testing for the initial time. Persistent HR-HPV infection was determined as at least two consecutive visit with the same genotype as the initial testing. If HR-HPV tested negative or with different HR-HPV genotypes than the initial testing in the next two periods, it was considered as transient HR-HPV infection (detailed in Additional file 2: Table S1). Single HR-HPV infection referred to the infection with single genotype in per HR-HPV testing, while multiple HR-HPV infection referred to the infection with two or more genotypes at least one HR-HPV testing.

### HR-HPV testing and genotyping

HPV testing was performed using the HR‐HPV typing test kit (Shanghai ZJ Bio‐Tech Co, Ltd.) according to the manufacturer’s instructions in an AGS 4800 real-time PCR System. Four fluorescent channels of different reaction tubes correspond to specific HPV types and internal control (IC) (FAM: HPV16, 18, 45, 39; VIC: HPV 56, 52, 82, 51; Red 610: HPV 31, 58, 33, 59; Cy5: HPV 68, 35, 66, and IC). The positive results were with a cycle threshold value (CT value) ≤ 38 and with a typical "S-shape" amplification curve.

### SNPs genotyping

Polymerase chain reaction-restriction fragment length polymorphism (PCR–RFLP) was performed to identify the *TLR4* and *TLR9* gene polymorphisms. We designed new primers with the software Primer Premier 6 and synthesized by Sangon Biotech Co., Ltd. The primer sequences as followed, rs10116253 F: 5′-TGTGATGATTAGGGCTGAA-3′, rs10116253 R: 5′-GTGGACTGGGCACAAACT-3′; rs1927911 F: 5′-CATGTGCCTCTGAACTTA-3′, rs1927911 R: 5′-CATGCACTCTAAAGATTTC-3′; rs10759931 F: 5′-ACATTGGTAGCACCAGAGTC-3′, rs10759931 R: 5′-ATTTCCCTTACTTCCTCATT-3′; rs187084 F: 5′-TCCCAGCAGCAACAATTCATTA-3′, rs187084 R: 5′-CTGCTTGCAGTTGACTGTGT-3′; rs352140 F: 5′-CCAGGTAATTGTCACGGAGA-3′, rs352140 R: 5′-TCTCGCAGGCAGTCAATG- 3′. The details of primers and specific restriction enzymes were shown in Additional file 3: Table S2. PCR was carried out in a total reaction volume of 25 μl with 15.875 μl ddH_2_O, 2.5 μl 10 × buffer (Mg^2+^) (Takara), 2.0 μl dNTPs (Takara), 1.25 μl forward and reverse primers (20uM) (Sangon Biotech), 0.125 μl Taq DNA polymerase (5 U/L) (Takara), and 2 μl DNA extraction. PCR under the following conditions: initial denaturation of 95 °C for 5 min, 35 cycles of 98 °C for 10 s, 55 °C or 60 °C for 45 s, and 72 °C for 1 min. The PCR products were digested by specific restriction enzymes, and then analyzed on 3% agarose gel electrophoresis stained by GelRed nucleic acid dye (Biotium).

### Statistical analysis

All statistical analyses were performed with SPSS software version 25.0. The continuous variables compared between case and control group using Student’s t-test. Comparison between categorical variables was performed by the χ^2^ test. The distribution of HR-HPV infection status and time for HR-HPV clearance with respect to the different *TLR4/TLR9* genotypes and alleles were furtherly analyzed. Logistic regression analysis was used to determine the association of *TLR4/TLR9* genotypes and alleles with HR-HPV infection status by computing the adjusted odds ratios (ORs) and 95% confidence intervals (CI). Hardy–Weinberg equilibrium (HWE) and frequencies of haplotype were determined by SNPstats program (http://bioinfo.iconcologia.net/SNPstats). The time for HR-HPV clearance was plotted by the Kaplan–Meier method and compared by log-rank test to analyze its relationship with *TLR4/TLR9* genotypes (the time for HR-HPV clearance was defined as the time span between the first HR-HPV positive testing and the subsequent negative or different genotypes testing). A two-tailed P < 0.05 was considered statistically significant.

## Results

### Age distribution and HR-HPV infection in the enrolled subjects

A total of 269 HPV-positive women were included in this case–control study (transient infection:103, persistent infection: 166; single infection:160, multiple infection:109). The average age (mean ± SD) of patients with persistent HR-HPV infection (46.89 ± 9.72) was higher than that of patients with transient infection (41.55 ± 8.65) (*P* < 0.001), as well as multiple HR-HPV infection (46.62 ± 11.02) was higher than single infection (43.64 ± 8.25) (*P* = 0.018). Among single HR-HPV infection, the most prevalent genotypes were HPV52 (51/160 31.9%), HPV16 (21/160 13.1%) and HPV58 (17/160 10.6%). Among multiple HR-HPV infection, the most prevalent genotypes were HPV52 (50/109 45.9%), HPV58 (47/109 43.1%) and HPV16 (28/109 25.7%) (Table 1).

**Table 1:** Age distribution and HR-HPV infection prevalence of study subjects

Variables	Transient HR-HPV infection (n = 103)	Persistent HR-HPV Infection (n = 166)	P1	Single HR-HPV infection (n = 160)	Multiple HR-HPV infection (n = 109)	P2
Age (years)						
Mean ± SD	41.55 ± 8.65	46.89 ± 9.72	< 0.001	43.64 ± 8.25	46.62 ± 11.02	0.018
18–29	12 (11.7)	14 (8.4)		12 (7.5)	14 (12.8)	
30–39	27 (26.2)	20 (12.0)		33 (20.6)	14 (12.8)	
40–49	48 (46.6)	66 (39.8)		79 (49.4)	32 (29.4)	
≥ 50	16 (15.5)	66 (39.7)	< 0.001	36 (22.5)	49 (45.0)	< 0.001
HR-HPV genotypes						
HPV16	13 (12.6)	11 (6.6)		21 (13.1)	28 (25.7)	
HPV18	5 (4.9)	9 (5.4)		12 (7.5)	12 (11.0)	
HPV52	16 (15.5)	60 (36.2)		51 (31.9)	50 (45.9)	
HPV58	9 (8.7)	31 (18.7)		17 (10.6)	47 (43.1)	
HPV68	4 (3.9)	3 (1.9)		3 (1.9)	24 (22.0)	
HPV66	5 (4.9)	5 (3.1)		8 (5.0)	17 (15.6)	
HPV56	4 (3.9)	3 (1.9)		4 (2.5)	17 (15.6)	
HPV31	5 (4.9)	3 (1.9)		7 (4.3)	7 (6.4)	
HPV33	2 (1.9)	4 (2.4)		4 (2.5)	6 (5.5)	
HPV35	2 (1.9)	2 (1.2)		3 (1.9)	5 (4.6)	
HPV45	8 (7.8)	5 (3.1)		3 (1.9)	2 (1.8)	
HPV82	2 (1.9)	1 (0.1)		0	1 (0.1)	
HPV39	0	0		9 (5.6)	11 (10.1)	
HPV51	12 (11.7)	7 (4.2)		15 (9.4)	12 (11.0)	
HPV59	2 (1.9)	8 (4.8)		3 (1.9)	15 (13.8)	
Multiple genotypes	14 (13.6)	14 (8.5)		NA	NA	

HR-HPV: High risk-human papillomavirus; SD: Standard deviation; NA: Not applicable

P1: Transient HR-HPV infection VS. Persistent HR-HPV infection

P2: Single HR-HPV infection VS. Multiple HR-HPV infection

### The association of *TLR4/TLR9* polymorphisms with persistent and multiple HR-HPV infections

The frequencies of genotype and allele and their association with persistent and multiple HR-HPV infections were shown in Tables 2 and 3. Among the study groups, the distribution of genotypes was in accordance with HWE (*P* > 0.05). Two SNPs, rs187084 and rs352140 of *TLR9*, were in significant association with persistent and multiple HR-HPV infections after they were adjusted by age and HR-HPV genotypes. Compared with wild-type genotype, *TLR9* rs187084 heterozygous and mutant genotypes increase the risk of persistent (CT vs. TT, OR = 1.98, 95% CI = 1.08–3.64,* P* = 0.028; CT+CC vs. TT, OR = 2.07, 95% CI = 1.17–3.65, *P* = 0.012) and multiple (CT vs. TT, OR = 2.01, 95% CI 1.17–3.45, *P* = 0.011; CT+CC vs. TT, OR = 1.99, 95% CI = 1.19–3.31, *P* = 0.008) HR-HPV infections. *TLR9* rs352140 heterozygosity and mutant genotype also significantly increased the risk of HR-HPV persistent infection (CT vs. TT, OR = 2.01, 95% CI = 1.09–3.68, *P* = 0.024; CT+CC vs. TT, OR = 1.97, 95% CI =1.12–3.47, *P* = 0.018). Without adjusted by age, *TLR9* rs352140 mutant genotype was associated with multiple HR-HPV infections in the dominant model (CT+ TT vs. CC; OR = 1.71, 95% CI =1.04–2.81,* P* = 0.036). The mutant allele of *TLR9* rs187084 and rs352140 increase the risk of persistent (rs187084: C vs. T; OR = 1.70, 95% CI = 1.12–2.58, *P* = 0.013; rs352140: T vs. C; OR = 1.56, 95% CI = 1.03–2.37, *P* = 0.037) and multiple (rs187084: C vs. T; OR = 1.54, 95% CI = 1.07–2.22, *P* = 0.020; rs352140: T vs. C; OR = 1.44, 95% CI = 1.01–2.06,* P* = 0.047) HR-HPV infections.

**Table 2:** The genotype and allele frequencies of *TLR4/TLR9* SNPs and their associations with persistent HR-HPV infection

Gene	SNPs	Genotype/Allele	Transient HR-HPV infection (n%)	Persistent HR-HPV infection (n%)	OR (95% CI)	P-Value ^a^
TLR4	rs10116253	TT	36 (35.0)	60 (36.1)	1.00	
		CT	47 (45.6)	81 (48.8)	0.98 (0.53–1.82)	0.950
		CC	20 (19.4)	25 (15.1)	0.75 (0.32–1.73)	0.746
		CT+CC	67 (65.0)	106 (63.9)	0.91 (0.51–1.64)	0.764
		T	119 (57.8)	201 (60.5)	1.00	
		C	87 (42.2)	131 (39.5)	0.89 (0.60–1.32)	0.563
	rs1927911	CC	35 (34.0)	60 (36.1)	1.00	
		CT	47 (45.6)	82 (49.4)	0.98 (0.53–1.82)	0.940
		TT	21 (20.4)	24 (14.5)	0.60 (0.26–1.39)	0.234
		CT+TT	68 (60.0)	106 (63.9)	0.87 (0.48–1.55)	0.628
		C	117 (56.8)	202 (60.8)	1.00	
		T	89 (43.2)	130 (39.2)	0.82 (0.55–1.22)	0.323
	rs10759931	AA	37 (35.9)	59 (35.5)	1.00	
		AG	48 (46.6)	81 (48.8)	0.98 (0.53–1.82)	0.960
		GG	18 (17.5)	26 (15.7)	1.01 (0.44–2.35)	0.980
		AG+GG	66 (64.1)	107 (64.5)	0.99 (0.57–1.76)	0.973
		A	122 (59.2)	199 (59.9)	1.00	
		G	84 (40.8)	133 (40.1)	1.00 (0.67–1.49)	0.996
TLR9	rs187084	TT	55 (53.4)	61 (36.7)	1.00	
		CT	37 (35.9)	80 (48.2)	1.98 (1.08–3.64)	**0.028**
		CC	11 (10.7)	25 (15.1)	2.37 (0.97–5.83)	0.059
		CT+CC	48 (46.6)	105 (63.2)	2.07(1.17–3.65)	**0.012**
		T	147 (71.4)	202 (60.8)	1.00	
		C	59 (28.6)	130 (39.2)	1.70 (1.12–2.58)	**0.013**
	rs352140	CC	55 (53.4)	62 (37.4)	1.00	
		CT	36 (35)	80 (48.2)	2.01 (1.09–3.68)	**0.024**
		TT	12 (11.6)	24 (14.4)	1.87 (0.77–4.52)	0.167
		CT+TT	48 (46.6)	104 (62.6)	1.97 (1.12–3.47)	**0.018**
		C	146 (70.9)	204 (61.4)	1.00	
		T	60 (29.1)	128 (38.6)	1.56 (1.03–2.37)	**0.037**

SNP: Single nucleotide polymorphism; OR: Odds ratio; *TLR4*: Toll-like receptor 4; *TLR9*: Toll-like receptor 9. Bold values are statistically significant

^a^Adjusted for age and HR-HPV genotypes

**Table 3:** The genotype and allele frequencies of *TLR4/TLR9* SNPs and their associations with multiple HR-HPV infections

Gene	SNPs	Genotype/Allele	Single HR-HPV infection (n%)	Multiple HR-HPV infection (n%)	OR (95% CI)	P-Value ^a^
TLR4	rs10116253	TT	61 (38.1)	35 (32.1)	1.00	
		CT	75 (46.9)	53 (48.6)	1.14 (0.66–1.99)	0.635
		CC	24 (15.0)	21 (19.3)	1.69 (0.81–3.52)	0.160
		CT+CC	99 (61.9)	74 (67.9)	1.27 (0.75–2.13)	0.369
		T	197 (61.6)	123 (56.4)	1.00	
		C	123 (38.4)	95 (43.6)	1.27 (0.89–1.81)	0.186
	rs1927911	CC	60 (37.5)	35 (32.1)	1.00	
		CT	76 (47.5)	53 (48.6)	1.12 (0.64–1.94)	0.692
		TT	24 (15.0)	21 (19.3)	1.66 (0.80–3.45)	0.177
		CT+TT	100 (62.5)	74 (67.9)	1.24 (0.74–2.08)	0.417
		C	196 (61.3)	123 (56.4)	1.00	
		T	124 (38.7)	95 (43.6)	1.24 (0.87–1.77)	0.235
	rs10759931	AA	60 (37.5)	36 (33.0)	1.00	
		AG	76 (47.5)	53 (48.6)	1.08 (0.62–1.88)	0.776
		GG	24 (15.0)	20 (18.4)	1.54 (0.74–3.21)	0.252
		AG+GG	100 (62.5)	73 (67.0)	1.19 (0.71–1.99)	0.518
		A	196 (61.3)	125 (57.3)	1.00	
		G	124 (38.7)	93 (42.7)	1.16 (0.815–1.66)	0.403
TLR9	rs187084	TT	80 (50.0)	36 (33.0)	1.00	
		CT	61 (38.1)	56 (51.4)	2.01 (1.17–3.45)	**0.011**
		CC	19 (11.9)	17 (15.6)	1.91 (0.88–4.14)	0.100
		CT+CC	80 (50.0)	73 (67.0)	1.99 (1.19–3.31)	**0.008**
		T	221 (69.1)	128 (58.7)	1.00	
		C	99 (30.9)	90 (41.3)	1.54 (1.07–2.22)	**0.020**
	rs352140	CC	78 (48.8)	39 (35.8)	1.00	
		CT	63 (39.4)	53 (48.6)	1.64 (0.96–2.80)	0.070
		TT	19 (11.9)	17 (15.6)	1.69 (0.78–3.65)	0.180
		CT+TT	82 (51.2)	70 (64.2)	1.71 (1.04–2.81)	**0.036 **^**b**^
		C	219 (68.4)	131 (60.1)	1.00	
		T	101 (31.6)	87 (39.9)	1.44 (1.01–2.06)	**0.047 **^**b**^

SNP: Single nucleotide polymorphism; OR: Odds ratio; *TLR4*: Toll-like receptor 4; *TLR9*: Toll-like receptor 9. Bold values are statistically significant

^a^Adjusted for age

^b^Not adjusted

However, no significant differences in *TLR4* SNPs (rs10116253, rs1927911, rs10759931) genotype and allele frequencies were found between the persistent and transient HR-HPV infection groups, as well as between the single and multiple HR-HPV infection groups (all *P* > 0.05).

### Linkage disequilibrium and haplotype analysis

Our data showed that *TLR4* and *TLR9* SNPs candidate loci were in strong linkage disequilibrium (LD), respectively. The most frequencies of haplotypes T-C and C-T of *TLR9*(rs187084-rs352140) in the study population were 63.93% and 34.00%, respectively, and the most frequencies of haplotypes T-C-A and C-T-G of *TLR4* (rs10116253-rs1927911-rs10759931) were 58.73% and 39.58%, respectively. Compared with the *TLR9* haplotypes T-C, carriers with the C-T haplotypes were more susceptible to persistent and multiple HR-HPV infections (*P*1 = 0.026; *P*2 = 0.048) (Table 4). However, no significant association was observed between *TLR4* haplotypes and HR-HPV infection status.

**Table 4:** The association of *TLR4/TLR9* haplotypes with HR-HPV infection status

SNPs	Haplotype	Frequencies			P1^a^	P2^b^
		Overall (%)	Transient infection/Persistent infection (%)	Single infection/multiple infection (%)		
*TLR4* rs10116253-rs1927911-rs10759931	T-C-A	58.73	56.80/59.94	60.30/56.42		
	C-T-G	39.58	40.27/39.16	37.49/42.66	0.67	0.22
	C-T-A	0.75	NA	0.63/0.92	0.12	0.49
	T-C-G	0.38	NA	NA	NA	NA
	T-T-G	0.19	NA	NA	NA	NA
	C-C-G	0.19	NA	NA	NA	NA
	T-T-A	0.18	NA	NA	NA	NA
*TLR9* rs187084-rs352140	T-C	63.93	70.38/59.93	68.12/57.78		
	C-T	34.00	28.15/37.64	30.62/38.97	**0.026**	**0.048**
	C-C	1.13	0.49/1.52	0.32/2.32	0.24	**0.042**
	T-T	0.94	0.97/0.92	0.94/0.94	0.46	1

SNP: Single nucleotide polymorphism; *TLR4*: Toll-like receptor 4; *TLR9*: Toll-like receptor 9; NA: Not applicable. Bold values are statistically significant

P1: Transient infection VS. Persistent infection; P2: Single infection vs. multiple infections

^a^Adjusted for age and HR-HPV genotypes

^b^Adjusted for age

### Kaplan–Meier analysis

**Fig. 2:**
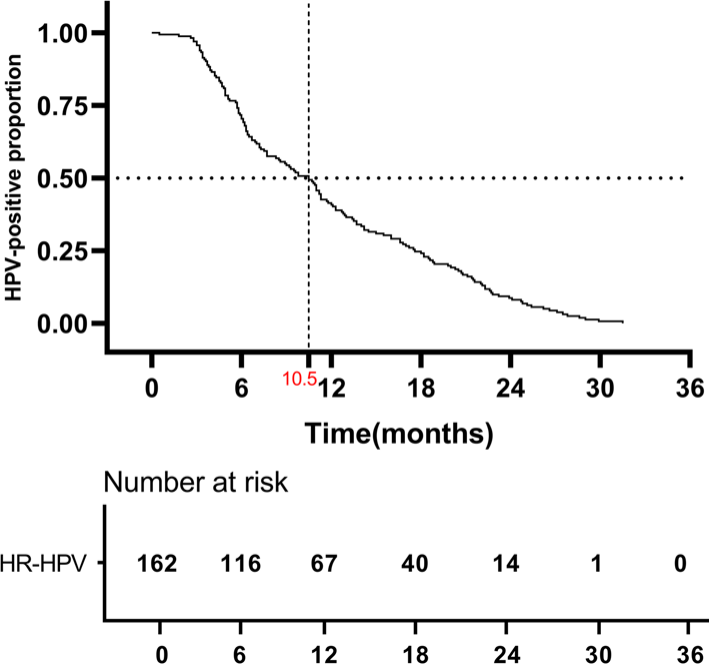
Time to clearance of prevalent detected individuals HR-HPV infection. The median HR-HPV clearance time was marked in red. The number of patients at risk were listed below the curve

**Fig. 3:**
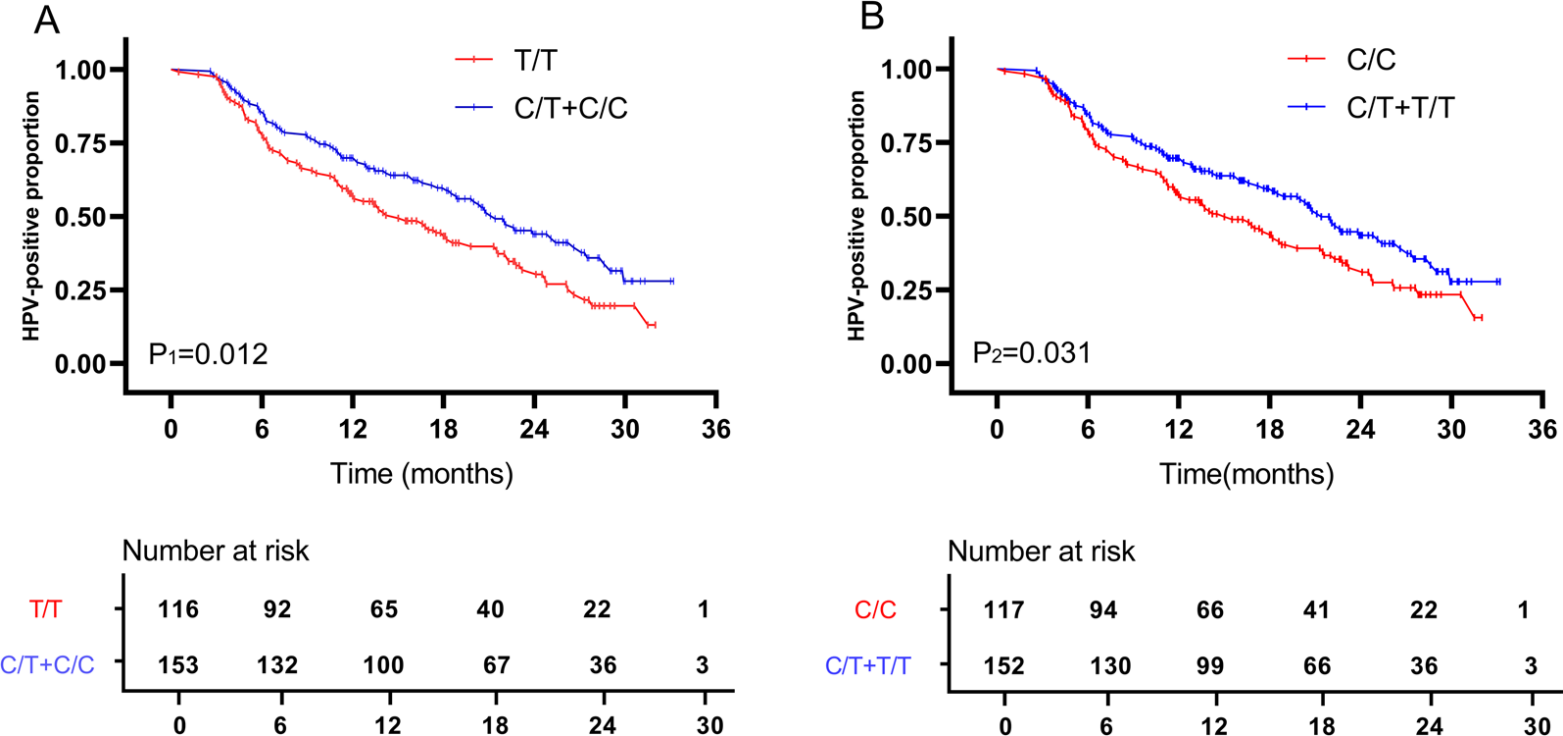
Kaplan–Meier curve for time to HR-HPV clearance in patients with different *TLR9* SNPs genotypes (**A**: rs187084; **B**: rs352140). The differences were determined by the log-rank test. The number of patients at risk were listed below each curve

Kaplan–Meier analysis was performed to reveal the correlation between HR-HPV clearance and *TLR4/TLR9* polymorphisms. The median follow-up period was 24.5 months (range, 2.6–33.2 months). There were 162 (60.22%) patients to be HR-HPV negative or tested with different HR-HPV genotypes than the initial test at the end of our follow-up. The median HR-HPV clearance time was 10.5 months in those 162 patients (Fig. 2). Compared with wild-type of *TLR9* rs187084 and rs352140 carrier, patients carrying CC+CT genotypes of rs187084 and CT+TT genotypes of rs352140 take longer duration to clear HR-HPV infection (*P*1 = 0.012; *P*2 = 0.031) (Fig. 3). However, negative results were found between *TLR4* SNPs genotypes that compared by log-rank test (Additional file 1: Fig. S1).

## Discussion

If failure to clear the HR-HPV infection and exposure to novel infection continues, women will be prone to accumulate different HR-HPV genotypes and a higher prevalence of multiple HR-HPV genotype infection, ultimately increasing the risk of HR-HPV persistent infection [32]. Moreover, HR-HPV has developed several mechanisms to avoid host immune response, which was important for viral persistent infection [17]. Genetic factors were responsible for the susceptibility and clinical outcomes of infectious diseases. Genetic mutation in *TLR4/TLR9* may alter the expression or function of encoded proteins and their association with susceptibility to viral infections has been reported in different populations [11]. However, there are only a few studies available investigating the role of *TLR4/TLR9* gene polymorphisms in the natural history of HR-HPV infection. In this study, *TLR4* rs10116253, rs1927911, rs10759931 and *TLR9* rs187084, rs352140 were genotyped in 269 HR-HPV-positive women. We found *TLR9* rs187084 and rs352140 mutant allele carriers took longer duration to clear HR-HPV infection, likely to be at an increased risk of persistent and multiple HR-HPV infections.

*TLR9* gene is located on chromosome 3p213, in which rs187084 (located in the promoter region) and rs352140 (located in the exon) are 2 common SNPs. It was reported that rs187084 TT genotype and T allele were associated with HPV infection in Mexican women [23]. In addition, the study among Indian women found heterozygous genotype CT of rs352140 increasing the risk of HPV16 and HPV18 infection [24]. Above studies only partly showed that rs187084 and rs352140 influenced HPV susceptibility, but never explicitly explained their role in the natural history of HPV infection. In this study, our data indicated that mutant genotypes (rs187084: CT and CT+CC; rs352140: CT and CT+TT), mutant allele (rs187084:C; rs352140: T), and haplotype C-T of rs187084 and rs352140 were correlated with HR-HPV persistent infection. Similarly, Oliveira et al. [27] investigated the role of SNP rs5743836 in the promoter region of the *TLR9* gene and its association with the risk of acquisition and clearance of type-specific HPV infection using a case–control sampling strategy within the Ludwig–McGill Cohort Study, whose data showed no consistently significant associations between *TLR9* rs5743836 polymorphism and HPV clearance or persistence, probably due to the limited sample sizes. In other studies, the association of rs187084 with HBV infection and rs352140 with HCV spontaneous clearance were also reported to be absent [33–35]. However, Loganathan et al. [36] reported that the heterozygous variant genotype of TLR9 rs352140 (CT) favored the persistence of helicobacter pylori infection, which was in good agreement with our results of HR-HPV persistent infection. Moreover, Bharti et al. [37] stimulated PBMCs from individuals with the different rs187084 genotypes and observed significantly lower interferon-gamma (IFNγ) and tumor necrosis factor-alpha (TNFα) mRNA expressions in the individuals with the TC genotype than in those with the TT genotype. The cytokines, IFNγ and TNFα, play pivotal roles in macrophage activation during the elimination of intracellular HPV [38]. Therefore, it seems to imply rs187084 CT carriers are with increased risk of persistent HR-HPV infection compared to TT carriers.

As the definition of persistent HPV infection is ambiguous, there may be selection bias in our subgroups. Kaplan–Meier time to clearance analysis better reflects the natural history of HR-HPV. We performed Kaplan–Meier analysis to clarify whether *TLR4* and *TLR9* polymorphisms were associated with HR-HPV spontaneous clearance. Similar to other studies [4–7, 39], our data showed most HPV infections (148/162, 91.36%) were cleared within 2 years and the median HR-HPV clearance time was 10.5 months. Therefore, regular HPV re-examination over 2 years is necessary for HR-HPV-positive population. Moreover, our data showed that mutant allele carriers of *TLR9* rs187084 and rs352140 presented a significantly longer duration time for HR-HPV clearance compared to wild-type patients, which was consistent with the conclusions from our case–control studies. Our findings suggest that women with different *TLR9* SNPs genotypes might undergo different time for HR-HPV infection, and a personalized screening strategy might be a more efficient and economical choice.

The prevalence and distribution of single and multiple HPV infections vary widely worldwide and are affected by diverse factors [19, 40, 41]. In our study, we found that the majority of patients with multiple infections developed a single persistent infection of some HPV genotype during their history of infection. It seems to indicate a correlation between multiple infections and persistent infections. The study conducted by Kim et al. [19] reported patients with multiple HPV infections displayed persistent and longer duration of HPV infection compared to patients with single HPV infection. A possible mechanism is that multiple HPV infections may affect the immune status and lead to increased viral load [32]. Moreover, our study showed that the mutant genotypes/alleles of *TLR9* rs187084 and rs352140 increase the risk of HR-HPV multiple infections as well. It indicated that *TLR9* SNPs rs187084 and rs352140 may play the same roles in persistent and multiple HR-HPV infections. However, Pandey et al. [28] never observed a significant association between *TLR9* rs187084 and rs352140 polymorphisms and multiple HR-HPV infections in India. The reason for this may be the influence of different genetic background or other environmental factors, such as smoking, sexual behavior, and pathogens infection.

The chronic inflammation mediated by TLR4/iNOS and TLR4/MYD88/NF-κB signaling pathway was associated with HPV-related cervical cancer [42, 43]. It was reported that heterozygous genotype (CT) and mutant allele (T) of *TLR4* rs1927911 increased the risk of cervical cancer and HPV16/18 infection, while the role of SNP rs10116253 and rs10759931 in the HPV infection was not clear. In our study, we found that *TLR4* polymorphism was in lack of association with HR-HPV persistent and multiple infections. TLR4 could recognize lipopolysaccharide (LPS), a major component of the outer membrane of Gram-negative bacteria, as well as some envelope proteins from the virus [10]. Although it was reported that HPV16 L1 virus-like particles (VLP) activate B cells to induce CD4(+) T cell-independent humoral immune responses via TLR4- and MyD88-dependent signaling [44], whether HPV proteins were recognized by TLR4 was unknown. Therefore, TLR-mediated immune responses to HPV infection maybe not be directly activated by HPV proteins, but via other immune signaling pathways. We speculated that the specific anatomic location of the cervix and sexual activity increased the susceptibility of the cervix to microbial infection and exposed TLR4 to a high level of LPS environment [43], which led to TLR4 overexpress in cervical cancer tissue and facilitated the formation of a local immune microenvironment. A meta-analysis showed an intimate association between vaginal microecology and HPV infection [45]. Moreover, it was reported that *TLR4* polymorphisms were significantly associated with the susceptibility to *Chlamydia trachomatis* (CT), *Neisseria gonorrhoeae* (NG), and Trichomonas vaginalis (TV) infection [46–48]. Hence, the association of TLR4 expression and its gene polymorphisms with HR-HPV infection status needs to be further validated after excluding the influence of other bacterial and viral pathogens.

The main strengths of this study were that the subject was collected from a retrospective cohort that included patients followed-up for a long period of time (about 36 months), and HR-HPV infection status was confirmed with consecutive visits at least three times. Several limitations in our study should be considered as well. We were unable to collect more information of patients, such as smoking, use of condoms, and the number of sexual partners, which potentially have an impact on HR-HPV infection. The sample size was limited to some extent, we failed to analyze type-specific HR-HPV infection and its association with *TLR4/TLR9* polymorphisms. Only the superficial correlation between *TLR4/TLR9* polymorphisms and HR-HPV infection status was investigated in our research. Thus, we will expand our study populations for subgroup analysis and explore underlying mechanisms in further research.

## Conclusions

In conclusion, this study initially investigated the relationship between *TLR4* and *TLR9* gene polymorphisms and HR-HPV infection status in Chinese Han population. Our results suggested that the heterozygous genotypes and mutant alleles of *TLR9* rs187084 and rs352140 were associated with persistent and multiple HR-HPV infections, which maybe provide important information for further investigation of risk factors for HR-HPV infection and assessment of the HR-HPV infection status.

## Supplementary Information

**Additional file 1: Fig S1. **Kaplan–Meier curve for time to HR-HPV clearance in patients with different *TLR4* SNPs genotypes (A: rs10116253; B: rs1927911; C: rs10759931). The differences were determined by the log-rank test. The number of patients at risk was listed below each curve.

**Additional file 2: Table S1.** Definition of persistent and transient HR-HPV infections.

**Additional file 3: Table S2.** Primer sequences and restriction enzymes used for genotyping the studied SNPs.

## Data Availability

All data generated or analyzed during this study are included in this published article and its supplementary information files.

## Abbreviations

HPV: Human papillomavirus
HR-HPV: High-risk human papillomavirus
TLRs: Toll-like receptors
RT-PCR: Real-time polymerase chain reaction
PCR–RFLP: PCR-restriction fragment length polymorphism
CIN: Cervical intraepithelial neoplasia
CC: Cervical cancer
PRRs: Pattern recognition receptors
PAMPs: Pathogen-associated molecular patterns
CPG: Cytosine-phosphate-guanine
DCs: Dendritic cells
IFN: Interferon
MHC: Major histocompatibility complex
SNPs: Single nucleotide polymorphisms
LPS: Lipopolysaccharide
HCV: Hepatitis C virus
LIS: Laboratory information system
EMRs: Electronic medical records
IC: Internal control
ORs: Odds ratios
CI: Confidence intervals
HWE: Hardy–Weinberg equilibrium
LD: Linkage disequilibrium
IFNγ: Interferon-gamma
TNFα: Tumor necrosis factor-alpha
VLP: Virus-like particles
CT: Chlamydia trachomatis
NG: *Neisseria gonorrhoeae*
TV: Trichomonas vaginalis
