# Supplementary figures and images for: Association of TLR4 and TLR9 gene polymorphisms with cervical HR-HPV infection status in Chinese Han population
Source: BMC Infect Dis. 2023 Mar 13;23:152. doi: 10.1186/s12879-023-08116-z (PMC10012518; doi:10.1186/s12879-023-08116-z)

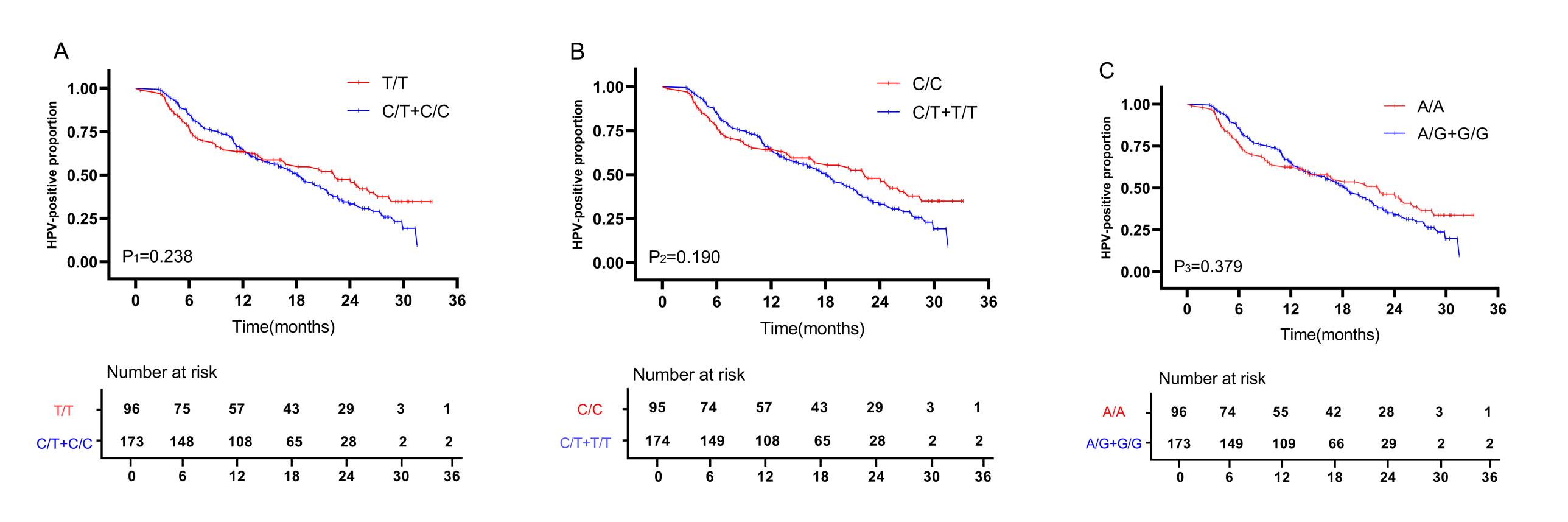

Supplement: Supplementary file 1 — Additional file 1: Fig S1. Kaplan–Meier curve for time to HR-HPV clearance in patients with different TLR4 SNPs genotypes (A: rs10116253; B: rs1927911; C: rs10759931). The differences were determined by the log-rank test. The number of patients at risk was listed below each curve. [file 12879_2023_8116_MOESM1_ESM.tif]
