# Supplementary material for: Association of TLR4 and TLR9 gene polymorphisms with cervical HR-HPV infection status in Chinese Han population
Source: BMC Infect Dis. 2023 Mar 13;23:152. doi: 10.1186/s12879-023-08116-z (PMC10012518; doi:10.1186/s12879-023-08116-z)
Supplement: Supplementary file 2 — Additional file 2: Table S1. Definition of persistent and transient HR-HPV infections. [file 12879_2023_8116_MOESM2_ESM.docx]

| **Table S1** Definition of persistent and transient HR-HPV infections. | | | |  |
| --- | --- | --- | --- | --- |
| Initial test | Time intervals | | |  |
|  | 0-12 months | 12-24 months | 24-36 months | **HR-HPV infection status** |
| P | P* | P* | P* | Persistent infection |
| P | P* | P* | N | Persistent infection |
| P | P* | N | P* | NA |
| P | P* | N | N | Transient infection |
| P | N | P* | P* | NA |
| P | N | P* | N | NA |
| P | N | N | P* | NA |
| P | N | N | N | Transient infection |
| P | P* | P* |  | Persistent infection |
| P | P* | N |  | NA |
| P | N | P* |  | NA |
| P | N | N |  | Transient infection |
| P | P* |  | P* | Persistent infection |
| P | P* |  | N | NA |
| P | N |  | P* | NA |
| P | N |  | N | Transient infection |
| P |  | P* | P* | Persistent infection |
| P |  | P* | N | NA |
| P |  | N | P* | NA |
| P |  | N | N | Transient infection |

P: HR-HPV positive; P*: Tested with same HR-HPV genotypes as the initial test; N: HR-HPV negative or tested with different HR-HPV genotypes than the initial test;

NA: Not applicable.
