# Supplementary material for: Association of TLR4 and TLR9 gene polymorphisms with cervical HR-HPV infection status in Chinese Han population
Source: BMC Infect Dis. 2023 Mar 13;23:152. doi: 10.1186/s12879-023-08116-z (PMC10012518; doi:10.1186/s12879-023-08116-z)
Supplement: Supplementary file 3 — Additional file 3: Table S2. Primer sequences and restriction enzymes used for genotyping the studied SNPs. [file 12879_2023_8116_MOESM3_ESM.docx]

| Table S2 Primer sequences and restriction enzymes used for genotyping the studied SNPs. | | | | |
| --- | --- | --- | --- | --- |
| **ID** | **Primer sequences (5′→3′)** | **Annealing temperature** | **Restriction enzyme used**  **(temperature /time)** | **Product size, bp** |
| ***TLR4***  rs10116253  rs1927911  rs10759931 | Forward: 5′-TGTGATGATTAGGGCTGAA- 3′  Reverse:5′- GTGGACTGGGCACAAACT- 3′  Forward: 5′-CATGTGCCTCTGAACTTA- 3′  Reverse: 5′- CATGCACTCTAAAGATTTC- 3′  Forward: 5′-ACATTGGTAGCACCAGAGTC- 3′  Reverse: 5′- ATTTCCCTTACTTCCTCATT- 3′ | 60℃  55℃  55℃ | BsmAI (55℃/1h)  StyI (37℃/1h)  KpnI (37℃/1h) | **TT**:312+165 **CC**:477  **CT:** 477+312+165  **CC:**156+62 **TT**:218  **CT:** 218 +156+62  **GG**:260+183 **AA:**443  **GA:** 443+183+260 |
| ***TLR9***  rs187084  rs352140 | Forward: 5′- TCCCAGCAGCAACAATTCATTA- 3′  Reverse: 5′- CTGCTTGCAGTTGACTGTGT- 3′  Forward: 5′- CCAGGTAATTGTCACGGAGA- 3′  Reverse: 5′- TCTCGCAGGCAGTCAATG- 3′ | 60℃  60℃ | AflII (37℃/1h)  BstUI (60℃/1h) | **TT:**327+172 **CC:** 499  CT:499 +172+327  **CC**:361+113 **TT**:474  **CT:**474+361+113 |

SNP: Single nucleotide polymorphism; bp: base pair; *TLR4*: Toll-like receptor 4; *TLR9*: Toll-like receptor 9.
